# Management of a High-Performing Mental Health Recovery Research Group

**DOI:** 10.3390/ijerph18084007

**Published:** 2021-04-11

**Authors:** Mike Slade

**Affiliations:** School of Health Sciences, Institute of Mental Health, University of Nottingham, Triumph Road, Nottingham NG7 2TU, UK; m.slade@nottingham.ac.uk

**Keywords:** mental health, recovery, research management, productivity, lived experience

## Abstract

A personal perspective is given on the processes involved in managing and sustaining a high-performing mental health recovery research group. The broader context of scholarship in the United Kingdom is outlined, in which academic productivity is commodified specifically in relation to peer-reviewed journal papers. Four leadership choices in developing a high-performing research group are discussed: optimal group size; sharing the workload; maintaining a programmatic focus; and performance expectations. Approaches to maximising innovation are identified, including emotional and intellectual engagement of team members, working with diverse stakeholders and convening communities of practice. We use a highly managed approach to publications from inception to acceptance, which is described in detail. The use of these approaches is illustrated in relation to the Recovery Research Team which was formed in 2009. Specific recovery-related issues covered include demonstrating the ability to develop a significant recovery research portfolio (our four current large [>UK£2 m] studies relate to recovery narratives, global mental health peer support work, digital interventions and Recovery Colleges); the positive implications of actively recruiting researchers with mental health lived experience; how performance issues are managed; our approach to involving lived experience co-authors in papers; and our decision to conduct mixed-methods rather than solely qualitative studies.

## 1. Introduction

In any job, doing well at ‘feeding the beast’ by meeting key performance indicators has many advantages. Job satisfaction is higher, especially where organisational and personal goals are aligned. Promotion prospects are enhanced. Negotiating power to decline invitations for less desired roles is increased. Standing among colleagues and in the employing organisation is higher. Finally, your chances of being fired are somewhat lower.

What are the key performance indicators in mental health research? For university-based academics, domains of success include research income, publications, teaching, knowledge mobilisation, societal and cultural impact, and academic administration. For clinical academics, this daunting list also includes clinical practice and clinical education. The balance between these domains of course varies for different roles, but also as systemic priorities evolve. For example, since 2017, the United Kingdom (UK) has been developing a Knowledge Exchange Framework to ‘*benchmark how well universities are doing at fostering knowledge sharing and research commercialisation*’ (p. 79) [1]. This has led to a recent stronger focus on knowledge mobilisation as a role expectation. Clearly a successful research career will pivot over time to maximise performance across existing and emerging domains. However, as few of us can be internationally excellent in all domains, prioritisation is needed. For individuals pursuing a research career, the two most central and foundational domains of success are research income and publications.

Grants matter. Whilst not discounting the physicist Ernest Rutherford’s advice ‘*we have run out of money—it is time to start thinking*’, in many areas of mental health research, it is very difficult to produce credible new knowledge without significant funding. Grants bring together experts, are front loaded with intellectual input in order to pass the funder review process which reduces waste when conducting the research, and may produce more methodologically rigorous findings than unfunded ‘own-account’ studies [2]. Own-account research has some advantages such as potentially being more innovative, but a recent qualitative study identified that ‘unfunded’ really means ‘self-funded’, in researcher time and effort [3]. Trying to conduct under-funded research can adversely impact on quality of life. Whilst unfunded research may sometimes be necessary, most of us work hard enough already so I tend to view own-account research as an option of last resort. Better is to obtain resources then do the work. To set the context, I created my research group—the Recovery Research Team—in 2009. The team is completely grant funded apart from my position. We currently hold UK£12.1 m of research funding including four £2 m–£4 m grants: NEON on recovery narratives, RECOLLECT on Recovery Colleges, Nottingham BRC on digital interventions and UPSIDES on global mental health peer support work. Information about all our studies is at www.researchintorecovery.com (accessed on 10 April 2021).

Publications also matter. They are a primary means of sharing scientific knowledge with the world. They meet the ethical obligation placed on researchers in the Declaration of Helsinki to make publicly available the results of their research on human subjects [4]. In neoliberal systems in which academic productivity is commodified, they are also the evidence of productivity. Academic publications come in many forms, including peer-reviewed journal papers and non-peer-reviewed grey literature, online (e.g., YouTube video, TED talk) and offline (e.g., PDF, printed journal), and video, audio or text-based modalities. However, in the UK, since 2001, the approximately five-yearly Research Assessment Exercise and more recently the Research Excellent Framework (REF) have deliberately focussed health research activities towards peer-reviewed data-based journal papers as the primary, if not only, type of important publication. As a result, many scholars who entered the UK academic community in the last two decades would be forgiven for thinking that peer-reviewed papers are the only knowledge production activity worth doing. By contrast, when I visit colleagues in other countries, their knowledge of my group’s work more often arises from having read one of our books (e.g., [5,6,7,8]) or booklets (e.g., [9,10,11,12]) or attended one of our conferences than from particular papers. Scholarship is a wider activity than any country-specific metric would suggest.

The Recovery Research Team is based in the UK, so to prosper, we have focussed on producing peer-reviewed publications as core business. Some journals now include a podcast section, e.g., Pages to Practice in *Psychiatric Services* and Talk medicine in *BMJ*. Video journals also exist, such as *JoVE* and *Video Journal of Clinical Research*. However, health research in the UK continues to almost exclusively use bibliometrics such as h-indices, citation metrics and journal impact factor, which are all based on traditional text-based papers published in academic journals. These metrics are used when evaluating promotion, grant and fellowship applications, and when demonstrating productivity in national research appraisal programmes such as REF. Scoring highly on these metrics is the academic system definition of playing the game well. For bench-marking purposes, the Recovery Research Team, which is generally viewed as a high-performing research group, published a mean of 21 peer-reviewed papers per year between 2015 and 2020.

**Figure 1:**
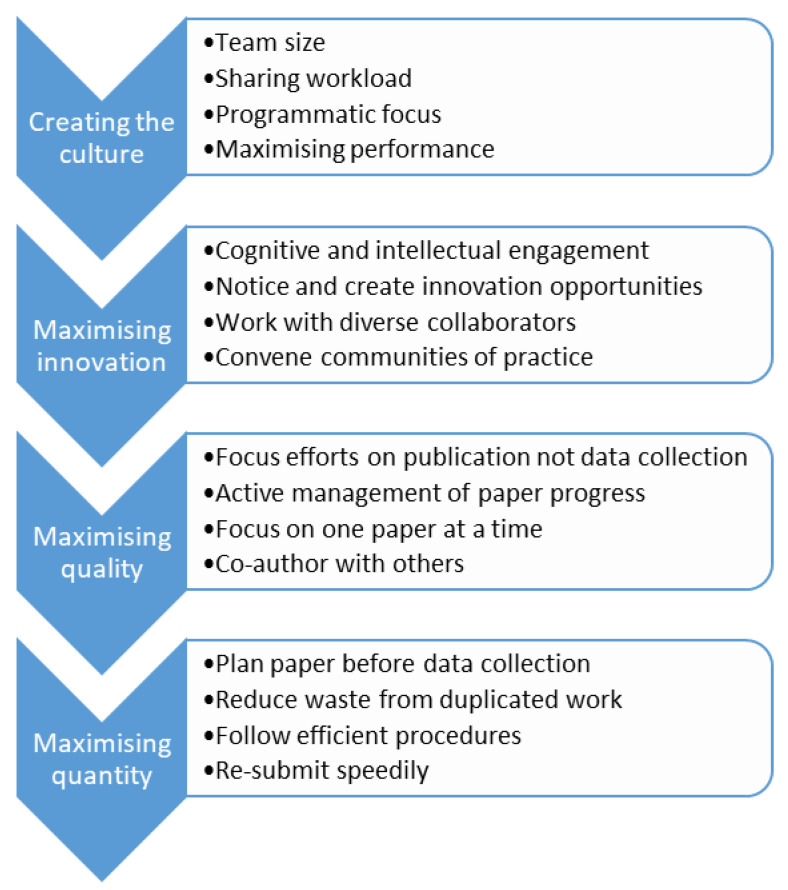
Recovery Research Team approach.

Our approach to managing high performance is now described. The aim of this approach is to improve, compared with less managed approaches, the innovation fostered by the team, the programmatic focus of the research, and the quality and quantity of outputs especially in relation to peer-reviewed academic publications. Our approach is summarised in Figure 1.

Each component of our approach is now discussed.

## 2. Creating the Culture

How does a leader create and sustain a high-performing research group? There are choices to be made. The first leader choice is size of group. I have headed research groups of varying sizes, from very small up to 60 people. My experience is that is it only possible for me to know the goals and something of the life situation of everyone in my team, and for them to know me with a closeness sufficient to inspire loyalty and discretionary effort, when the team size is at most around 20 people. Beyond that size, it is not possible to ‘know, love and inspire’ a team [13], and other leadership styles are needed. My preferred team size is therefore around 15; big enough to do meaningful work with economies of scale from cross-working whilst small enough to generate the emotional affiliation of a high-performing team without the emotional labour arising from the leadership role being too draining.

A second leader choice is sharing the workload. A good leader is really strong at delegating. This involves supporting less experienced researchers to grow in skills and confidence, rather than simply dumping jobs on people! Coaching and mentoring are key leadership skills, as is being seen to occasionally sacrificially ‘take one for the team’ rather than just retaining enjoyable tasks.

A third and related choice is in constantly balancing the need for individual autonomy with retaining a programmatic focus for the group as a whole. Our research focus is on recovery, social inclusion and wellbeing. Whilst I adopt a broad view of those issues, I retain and use veto rights when someone in my team wants to develop into an area too un-related to our core focus.

The final leader choice is how much to push people. On the one hand, all researchers in my team are on fixed-term contracts paid from publicly-funded grants. Obtaining best performance from contract researchers will maximise value for money for the tax payer, increase the scientific contribution of the research, and enhance the career prospects of the researchers. For example, we have had three five-year NIHR-funded programme grants—REFOCUS [14,15], NEON [16,17] and RECOLLECT [18,19]—and each post-graduate researcher employed full time for the whole programme can expect to emerge with 25 new grant-related academic papers in their CV.

On the other hand, people are not machines. Many team members live with significant challenges, including job precarity arising from fixed-term contracts, financial pressures due to gaps between jobs, housing insecurity caused by difficulties in getting on the property ladder, and fragile social networks following from employment mobility expectations. In our recovery-oriented research team, we actively seek to recruit team members with personal experience of mental ill health and recovery, and the majority of our team have lived experience. Whilst this adds great value to the quality of our work, any ongoing mental health-related difficulties can be another source of challenge.

How can the leader balance these considerations? We try to ensure individuals are making an informed choice to join our group, by foregrounding expectations in job descriptions about contributing to world-leading research, and actively recruiting individuals we believe to have future thought leadership potential. We also try to ensure that our team is mutually supportive, with regular (e.g., weekly) individual supervision meetings between each team member and his/her line manager which can include discussion of personal and professional development as well as study-related tasks and publications. We hold regular (e.g., monthly) team socials, celebrate birthdays, publicise achievements in internal team emails and as news items on our website (www.researchintorecovery.com/news/) (accessed on 10 April 2021), and I host Summer and Christmas get togethers at my home.

However, performance issues do arise. My view about the right balance starts with the stance that we are a professional research group and not a charity, so if someone is not able to achieve reasonable job performance expectations despite appropriate support then we will, if necessary, commence formal performance management processes AND that most performance challenges are better managed by retaining high expectations and co-developing workplace accommodations to support the individual to reach those standards. When I take the temperature of our team culture using 360 degree appraisals, common feedback from team members is “We like working here but you work us too hard”.

How do we maximise our recovery research output and impact? My approach has been directly informed by learning from numerous more experienced colleagues, and by publications about publication [20,21]. However, the following are all personal views. Key jobs of the research group leader are to maximise innovation and the quality and quantity of papers.

## 3. Maximising Innovation

To maximise innovation, researchers need to be emotionally and intellectually engaged. Emotional engagement arises from the study—and of course the goal is only to do research that matters—but also from the affiliation arising from the team culture. A litmus test I use is noting how team members respond when asked by others who they work for. In the complex ecology of our research group, there are many possible answers to this question, including the School, Faculty and University on the academic side, and the building, directorate and National Health Service organisation on the clinical side. My leadership goal is that everyone in my group identifies our Recovery Research Team as their primary affiliation.

Intellectual engagement, by contrast, is a cognitive task. My observation is that contract researchers involved in health research commonly misunderstand their role as a simplistic delivery of the pre-specified study protocol. Whilst this is necessary, it is not sufficient for innovation. The inter-related leadership challenges are supporting the person to understand that working in the knowledge economy involves actively contributing rather than passively complying, and supporting the growth of confidence and self-knowledge which allows the researcher to bring their own intellectual contribution to how the protocol is delivered. To meet these challenges, we have many study-specific and individual discussions about how to deliver the protocol, which allow us to identify when new and important questions emerge. For example, I was Principal Investigator (PI) for the REFOCUS study about recovery (2009–2014; researchintorecovery.com/refocus) (accessed on 10 April 2021). Our funded proposal protocol described an early planned systematic review of recovery-supporting interventions. When we came to do this review, we found we could not operationalise the construct of recovery. This led us to conduct an un-planned systematic review to conceptualise recovery, which developed the Connectedness, Hope, Identity, Meaning and Empowerment (CHIME) Framework. The resulting systematic review paper [22] is my most cited paper (1715 citations, Google Scholar, 20.3.21) and the CHIME Framework has become internationally influential as a theoretical foundation for understanding personal recovery [23]. The job of the leader is to be alive to and create these un-anticipated opportunities for original knowledge contributions.

It is possible to somewhat manage innovation using frameworks such as the quadruple helix approach [24]. For example, in the UK, significant research funding specifically to create innovation is awarded through the National Institute for Health Research *Invention for Innovation* programme. However, my experience is that innovation arises most commonly as an emergent and often un-expected property, and is more likely when networks of diverse stakeholders sponsor the innovation, when communities of practice inform and implement the change, and when no assumptions are made about which of research, policy and practice need to be the source of innovation. An example is the spread of Recovery Colleges [25], which now exist or are being developed in 22 countries [26]. The Recovery Research Team tries to create the context in which recovery-related innovation is more likely, in several ways:

- Our group is very multidisciplinary, comprising individuals (often with dual identity) with backgrounds in multiple academic disciplines and peer/survivor research, as well as a range of clinical professions.
- We put significant efforts into creating and sustaining international collaborations, for example by presenting at conferences and network meeting (mean 48 presentations per year 2015 to 2020), and we currently work with colleagues from around 40 countries.
- We deliberately work with diverse stakeholders, ranging from more conservative policy makers and professional representative groups to more critical survivor [27] and Mad Studies [28] perspectives, requiring significant time commitment and often testing our consensus-building skills.
- In non-pandemic times, we welcome visitors to our team and we regularly visit groups around the world, e.g., 23 visits by Recovery Research Team members to other groups in 2019.
- We use the convening power of our scientific credibility to create communities of practice, such as the Recovery Research Network (960 members from 38 countries; researchintorecovery.com/rrn) (accessed on 10 April 2021) and the biennial *Refocus on Recovery* conferences (2019 conference: 289 participants from 28 countries).

That does not mean we take every opportunity offered. For example, we do not accept funding from the pharmaceutical industry or other sources which might raise reputational concerns, e.g., the tobacco and arms industries. Similarly, in line with the ‘nothing about us without us’ civil rights value, I do not accept conference speaking invitations where there is not a lived experience speaker in an equivalent speaking slot.

## 4. Maximising Quality

Scientific quality requires active management. For all our large studies, we hold regular (e.g., fortnightly) separate meetings about tasks and publications. We separate these two functions in order to help early career researchers employed on the study to re-frame their core role as producing papers, hence viewing collecting and analysing the data as means not ends. This addresses the common difficulty of all the effort during the life of a study going into data collection, resulting in study papers being produced too slowly or not at all. Emphasising the benefits for their research CV is a good approach to help early career researchers grasp this distinction, as is the axiom “If you didn’t publish it, you didn’t do it”—akin to the clinical practice axiom “if you didn’t write it down, you didn’t do it”.

The writing process we use for papers led by our team is highly structured, and aims at every stage to reduce waste from un-used or duplicated work. We use a pre-formatted publications plan for each study, stored as a shared-access Microsoft Excel file. The headings used on the Planned Journal worksheet of the publications plan are shown in Table 1.

The categories and associated tasks for each paper status are shown in Table 2.

The publications plan includes other worksheets. The Published Journal worksheet includes the paper number, complete reference including doi, journal impact factor, PMID, PMCID, Registration (e.g., Prospero, ISRCTN), and Article Processing Charge to support budget management. The Planned Other and Published Other worksheets record information about conference presentations, non-peer-reviewed publications and print/social media articles. The Possible Papers worksheet is the memory bank for the study, recording ideas (expressed as a paper title), lead author and target journal for potential papers if resources allow. The Abandoned worksheet is where possible or planned publications are moved when effort is discontinued. The Author Information worksheet comprises the full list of all authors, comprising abbreviation (for use in other worksheets, e.g., ‘MSlade’), name, highest qualification, Affiliation, ORCID iD and email. Populating this for all likely authors at the start of the study is more time-efficient than obtaining journal-specific necessary author information at the point of submission.

Our expectation is that each researcher will, in addition to any papers which they have progressed to submission, be actively working on one paper and have a second paper identified as the next to work on. This means that each researcher is working on one paper at a time, whilst navigating submitted papers to acceptance and developing background thinking for their next paper. These are the papers in the Planned Journal worksheet. Other ideas for papers are recorded in the Possible Papers worksheet.

At each fortnightly publications meeting, we review all papers in the Planned Journal worksheet. Most air time is given to the papers currently being worked on, which are colour coded as amber. The whole team give intellectual input, and may organise future sub-team meetings to progress the paper. This has proved to be a valuable opportunity for sharing expertise and creating normative expectations. In general, we agree the target journal before any writing has occurred, and the lead author is expected to write fully in accordance with the relevant journal’s author guidelines from initial draft. This reduces waste and creates momentum, as some parts (e.g., title and author list, acknowledgements, funding statement) can be immediately written. Our general order of writing for pre-planned papers, such as those described in the funding bid where the rationale and methodology have been developed, is Methods and template Results such as un-populated tables, then Results, then Introduction and Discussion, and lastly Abstract and cover letter. The title and abstract are highly influential on decision-making by the editor, so should contain multiple keywords to increase findability, be clear and engaging to pique editor interest, and should emphasise the full importance of the study, such as in the quantity (‘first’, ‘largest’) or quality (‘first-in-field’, ‘most methodologically rigorous’) of the results or in highlighting significant or new implications for policy, practice or future research.

Progress with current submissions is briefly summarised, and where nothing has been heard about a submission for a long time (e.g., 3 months) we agree the corresponding author will politely contact the journal for a progress check. Papers which are next to work on are only discussed to agree title, author and target journal. When I am PI, I normally occupy last author role, allowing earlier career researchers to generate the first-author publications which can greatly enhance their career prospects. The exception is key papers, such as the main trial report, where it looks unusual if the PI is not in a first-author role (and because—of course—I also have to feed the beast). Most of our papers have multiple authors (2019 median 11 authors per paper, 2020 median 13 authors per paper), drawn from five groups shown in Table 3.

The order of groups in Table 3 reflects the amount of effort put into the paper, with the core writing team being the engine for producing a full draft, often informed by the wider internal research team and working closely with the LEAP co-authors. IAB and specific experts are only asked to comment on a relatively complete draft, reflecting our goals of minimising burden on busy collaborators and not wasting their time from commenting on unfinished or un-polished text. Authorship is only offered to individuals meeting the four International Committee of Medical Journal Editor (ICMJE) requirements (contribution; drafting; approval; accountability), and we have found the 15 potential contributor roles identified in the Contributor Roles Taxonomy (CRediT) framework [34] to be helpful in identifying the types of contributions made by each author.

Sometimes papers stall, such as when the lead author has left the team or an un-planned side-study cannot receive the intellectual input from the busy team it needs. In this case my leadership role is either to decide it should be abandoned or to take personal responsibility for progressing it from current draft to the point of submission. Almost always by this stage, a fair amount of work from the team has gone in, so I rarely advocate abandoning the paper. I typically get 5 to 8 papers per year ‘over the line’ from stalled to submission, and normally do not change the author order.

When a paper is submitted, after fulsome congratulations to the lead author and wider team, that paper is changed to green, the lead author’s red paper is changed to amber, and a new red paper is added from the Planned Journal worksheet.

## 5. Maximising Quantity

The above process is intended to streamline our approach to publishing papers. The quantity of outputs is increased when the process of publishing is well-managed, and the publications plan provides a vehicle for effective management. At all stages, the goal is increasing efficiency and reducing duplication. So, for example, the acknowledgements text required by funders is finalised early in the project and recorded in a worksheet in the publications plan for easy access. We all use the same bibliographic software (EndNote) when drafting manuscripts. Progress against agreed timelines as recorded in the publications plan is monitored in the two-weekly publications meeting, which is particularly helpful, if sometimes challenging, for researchers with a more pressure-prompted or deadline-driven working style.

We use two specific approaches to maximise quantity. First, we identify planned papers in parallel with developing the method. The advantage for qualitative studies is that the topic guide can then be highly focussed on the information needed for each planned paper. Our qualitative studies tend to have a large sample size compared to others, allowing the collection of rich data addressing more than one research question without ‘salami-slicing’ the data. For example, in NEON, one qualitative study involving 77 participants from under-researched groups led to publications about the impact of recorded recovery narratives [17], validation of a recovery narratives conceptual framework [35], post-traumatic growth and recovery [36] and institutional injustice [37]. For quantitative studies, the addition of one carefully-chosen standardised measure can justify a separate paper, as, for example, our use of a measure of pre-morbid IQ in a routine outcome measurement trial [38].

The second approach we use to maximise quantity is having a process to reduce the time between rejection and re-submission of a paper. Without such an approach the risk is that a rejected paper will never be re-submitted. Rejection is always a painful experience, and early career researchers may be particular affected due to repeated experiences of rejection which may activate impostor syndrome [39]. So the first goal when a rejection is received is to support the development of the resilience needed to prosper in an academic environment. One approach is to reinforce that the lead author is to be congratulated for doing the job of a professional researcher and not aiming too low. My personal heuristic is that I am probably getting the balance right between aiming too low and being un-realistically over-ambitious if I have around a 50% rejection rate on paper submissions.

What happens next? A rejection can be appealed, but in my experience this is almost never the right choice as it is very rarely successful and it generates extra work for the editor, which will inevitably influence their feelings the next time you submit a different paper to their journal. An exception might be where the rejection is based on one peer review which is of very poor quality.

The processes we then use are shown in Table 4.

In a professional research team, there should be a very rapid turnaround, e.g., one to two weeks, between rejection and re-submission. Co-authors have made their contribution so we do not bother them with constant updates about progress, generally emailing them with a copy of the submitted manuscript only when we start a new submission or to inform of acceptance.

Key parameters used by the Recovery Research Team, which may vary in other research groups, include

- Frequency of publication review meetings: we meet every two weeks but the optimal balance between talking about versus doing the work of writing may vary for different teams.
- We manage each study separately, so we have multiple publications plans which meets our need to focus on individual programmes of work, but means that integration between each study is a separate task.
- Where members of the wider internal research team are involved in only one paper, they attend just the part of the publications meeting about their paper, which reduces the time burden for them but also reduces their awareness of other papers.
- Our approach to multi-author publications may be less appropriate in other academic disciplines such as social science which have a tradition of fewer authors per paper.
- We prioritise reducing burden on less involved authors (groups 4 and 5 in Table 3), which may limit their ability to make early and formative intellectual contributions to papers.
- Whilst reducing time between rejection and re-submission is important, so too is allowing individuals to process emotions resulting from their submission being rejected, so expectations for a longer time period would be appropriate in less experienced research groups.

## 6. Conclusions

We have described our approach to managing a high-performing mental health recovery research group, up to the point of paper acceptance. Increasing the visibility, use and citation of the published paper is then the next task, which of course is a large topic in itself. Approaches we use include

- Publicising the paper in mainstream and social media [42], such as through press releases and policy briefings;
- Disseminating findings through our website researchintorecovery.com (accessed on 10 April 2021) (16,989 unique visitors from 125 countries in 2020);
- Circulating the paper to all Recovery Research Team members so they can cite it in their papers. Our programmatic approach of building bodies of knowledge rather than simply conducting un-related standalone projects justifies a high cross-citation of other papers from our group in new papers whilst avoiding citation manipulation [43];
- Storing the Author Final Draft (also known as Author Accepted Manuscript) on our institutional repository to ensure green open access requirements are met;
- Updating institutional staff pages and funder-mandated databases such as Researchfish;
- Presenting findings at conferences;
- Disseminating through networks, e.g., the monthly e-bulletin of the Recovery Research Network.

The intended advantages of our approach are maximising the operational efficiency, increasing the impact of the research, and ensuring long-term sustainability of the research group. Two potential disadvantages arise. First, our willingness to align with neoliberal values relating to commodification of scholarship means that we can easily be seen as part of the system by more critical mental health stakeholder groups, making partnership working more challenging. We address this by proactively engaging with many such groups with the aim of developing trusting relationships based on actual experience of working and learning together. Second, the centrality of the research group leader in overseeing processes can distract attention from the main topic focus on recovery. To address this we emphasise collective identity in numerous small ways, e.g., having a team name which is generic not ‘Mike Slade’s group’, listing our team alphabetically rather than by position on our website, employing first-person plural (we) not first-person singular (I) when describing our work, using participant badges containing just names without title (Dr, Prof, etc.) to de-emphasise status indicators at our events, not naming our knowledge products after individuals in the team, etc.

Our approach is more complex than less actively managed teams which, for example, collect the data then start the publication, or which do not use consistent processes or deadlines to support paper progress. To reduce complexity, we use shared and established procedures, rather than creating new approaches for each study. This makes delegation much more possible, so, for example, publications meetings can be held for a study without the Principal Investigator being present. A lot of organisational memory is needed about procedures, so we try to avoid having a large influx of new researchers in a short space of time. Our induction procedures are focused on creating team affiliation, so new joiners routinely meet with many other team members to become encultured into team procedures.

Two future developments can be identified. First, our approach has evolved through experience in the Recovery Research Team, and is not based on empirical evaluation. Recent advances in data science provide novel approaches to evaluating frameworks for optimising system performance and leadership through game theory [44], for example using fusion engines [45]. Future research might model the impact of varying key parameters for our approach on performance, e.g., number of papers, proportion of papers in higher impact factor journals, mean citation percentile for papers assessed using InCites reports. A second strand of future research relates to the transferability of our approach to other settings. Anecdotally we know that visitors to our group will often replicate some of our approaches when they return to their host group, but the cross-cultural relevance and impact of this knowledge transfer has not been evaluated.

Some recovery research groups take different approaches, for example prioritising hermeneutic and idiographic research primarily using qualitative methods in order to reflect the importance attached to lived experience. In contrast, the Recovery Research Team is intentionally mixed-methods, and our expertise includes randomised controlled trial leadership [14,46,47] and collaboration [48,49,50], as well as systematic reviews [51,52,53] and meta-analyses [54,55]. Whilst very aware of the limitations of the evidence-based medicine hierarchy [56,57], this orientation maximises the impact of the Recovery Research Team and has contributed to the ability to win large research awards and hence increased the sustainability of the group.

## Figures and Tables

**Table 1:** Planned Journal worksheet headings in study publications plan.

Heading	Rationale
Paper number	A unique number for each planned paper. All within-team emails include in the subject line the name of the study and the paper number, e.g., NEON #9. The cell is colour coded to make an at-a-glance overview of progress easy: Green = has been submitted, meaning the intellectual work has been completed, so the remaining task for the lead author is revising or re-submitting as necessary Amber = currently being written Red = not started but next to be worked on by the lead author
Provisional title	As close to a finalised title as possible—so not, e.g., ‘Qualitative study’. No hyphens in the title [29]. Use multiple keywords
1st author	The person who will write, or co-ordinate the writing of, the text to produce a complete first draft
Equal 1st author	An under-used option. Most journals allow this status, which is useful when authorship arrangements are complex such as in multi-site studies, and good for improving the CV of early career researchers
Other co-authors	Populate early with complete list in order. Use ‘name tbc’ (to be confirmed) when awaiting approval for involvement
Equal last author	Another under-used option which most journals allow and is useful for multi-site collaboration studies
Target journal	Identify target journal, normally before writing any of the paper
Status	Drop-down categories as shown in Table 2
Notes	Record target date for next milestone (e.g., ‘Full draft to co-authors by 22.1.22′) or submission history comprising submitted (‘S date’), revision requested (‘RR date’) and revision submitted (‘RS date’) details. Record previous rejections (‘No: journal name’) and future targets (‘Next: journal name’). For example: S 10.2.22, RR 11.6.22, RS 14.6.22, RR 1.7.22 No: Lancet Next: BMJ, American Journal of Psychiatry

**Table 2:** Status categories for papers.

Category	Meaning	Tasks
No data, not started	Decision made to progress paper. May be next-in-line paper for lead author, or may be a key paper (e.g., trial report) for writing later in the study	Finalise provisional title, confirm exact author list and target journal. Think about content
Awaiting editorial response	Editor of a specific journal has been contacted to ask if the paper would be of interest	Amend target journal if necessary
Data being collected	Paper has started	Write Methods section and blank Results section, e.g., prepare un-populated tables
Data collected, analysis not started	Data collection complete	Clean data, prepare database and data dictionary
Analysis underway	Data currently being analysed	Present emerging findings in publications meeting for team support with interpretation
Analysis complete	Data analysis finished	Populate Results section
1st draft underway	Full draft being written or co-ordinated by lead author	Write Introduction and Discussion, then Abstract. Add all journal-specific sections (e.g., author contributions) and follow author guidelines 100%
1st draft circulated locally	Full draft goes to internal team (groups 1 to 3 in Table 3) for comments, with e.g., 2 week deadline. Lead author revises.	Address all comments. Ensure all funder-required information is correct, e.g., Acknowledgements
Draft to all co-authors	Full draft goes to all authors including internal team again, with e.g., 3 week deadline.	Co-authors comment on full draft
Final draft underway	Lead author revises in light of all comments. Polish text. Work with specific co-author on individual section if needed. Write cover letter.	Create polished final version
Submission pending	Activate journal login and collate all materials needed for submission, e.g., recommended reviewers	Final look-over of full submission by study lead
Submitted	Lead author submits and confirms receipt	Submitted version sent to all co-authors
Revision requested	Journal requests revisions	Lead author informs internal team (but does not bother other authors), and co-ordinates revision
Revised version submitted	Lead author submits revision and confirms receipt	Lead author emails internal team with revised version to confirm re-submission
Re-drafting for next submission	Paper has been rejected	Lead author identifies new target journal, and re-drafts paper using process in Table 4
Accepted	Paper is accepted	Lead author emails all authors with Word and PDF versions of Author Final Draft, and confirms complete reference
Appealed	A rejection is being appealed	Lead author asks editor to re-consider their decision. Very rarely used

**Table 3:** Categories of author.

Category	Role
1. Core writing team	One or two lead authors who create the full draft
2. Wider internal research team	Make wider contributions, including to data collection, analysis, and commenting
3. Lived Experience Advisory Panel (LEAP)	Our larger studies all have a dedicated LEAP comprising 10 individuals with relevant lived experience, who work with us to ensure that lived experience informs every stage of our studies from design to dissemination. For most papers we identify two LEAP members to be co-authors. To note, other types of outputs such as chapters [30] and booklets [31] are led by the LEAP
4. International Advisory Board (IAB)	Our larger studies also all have an International Advisory Board (IAB) typically comprising 6 to 10 thought leaders from across the world, to ensure we are collaborating with best-in-field researchers. One advantage of having international authors is the increase in citation of the paper [32]
5. Specific experts	We may contact a specific world-leading expert to request their input in exchange for co-authorship, which has proved a valuable mechanism for developing new collaborations. Or we may involve a topic-specific expert to sense check the paper, e.g., a peer support worker (PSW) co-author in our global mental health PSW study [33]

**Table 4:** Process of re-submission after rejection.

**Step 1: Learning from the rejection**
If the paper was rejected by the editor without peer review, then read the editor feedback. Carefully. If the editor thought it was their type of paper but not of sufficient quality or innovation then consider re-submitting to a similar type of journal. Or if the editor thought it was not their type of paper, then re-consider the type of journal you are submitting to.
2. If the paper was rejected after peer review, then this is good news; the editor thought it worth considering for their journal so you are in the zone both in study quality and in choice of journal. Read the peer reviewer comments. Carefully and non-defensively. Their comments will inform your decision about the next journal. If they identify issues you can fix then fix them and learn from their feedback for your future writing endeavours. If they identify issues you cannot fix, e.g., sample size is too small, then ensure these limitations are noted in the manuscript and consider aiming lower down the journal impact factor food chain. 3. Sometimes editors offer transfer to an associated, generally lower impact factor, journal. Aspects to consider are: (a) how does the impact factor of the proposed journal differ from the planned next target journal? A big difference may mean that accepting the transfer would be aiming too low. (b) Can you afford the Article Processing Charge for the proposed journal? (c) Although it might initially seem like the easy option not involving any manuscript editing, sometimes there is significant re-writing into the new journal style requested of the author immediately the journal is transferred, so review the proposed new journal author guidelines before accepting the transfer.
4. In any event, and especially if the editor rejects without giving any useful feedback, re-read the components which the editor will skim in order to come to an initial view: cover letter, title, abstract and any journal-specific preface sections (e.g., What was known before and what this study adds; implications for practice). Are these in the right scientific voice, and as clear and interesting as possible?
**Step 2: Identify the new target journal**
Journal Impact Factor: although there are many issues with this metric (see the San Francisco Declaration on Research Assessment (DORA) [40]), impact factor currently reigns supreme in evaluating your research profile within health research, so aim high. Ensure you are using the Journal Citation Report (JCR) two-year metric [41] published by Clarivate Analytics (previously by Thomson Reuters), not the JCR five-year metric, not other credible but not-yet-accepted metrics such as Scimago Journal & Country Rank (SJR) (Www.scimagojr.com) (accessed on 10 April 2021), Altmetric (www.altmetric.com) (accessed on 10 April 2021) or Source Normalized Impact per Paper (SNIP)/CiteScore (www.scopus.com/sources) (accessed on 10 April 2021), and definitely not the bogus impact factor metrics used by predatory journals.
2. Audience: are you targeting the right type of journal readership? Will the take-home messages from your paper be both relevant and informative to this audience? 3. Article Processing Charge (APC): increasingly (with the Berlin Declaration [openaccess.mpg.de/Berlin-Declaration], Plan S [www.coalition-s.org] (accessed on 10 April 2021) and hybrid and Transformative Journals) journals levy APCs on authors—do budget limitations restrict your new target journal choices?
4. Academic discipline: if you plan to change from e.g., a psychiatry journal to a social sciences journal then that requires a different writing voice, with different assumptions about audience knowledge, which will be more work to revise than for a target journal from the same discipline.
5. Specific journal: identify a possible new target journal, if needed using advice from colleagues. Scan some recent paper titles published by that journal. Are they close enough in focus to your paper that the editor might assess your paper as in scope? Are they using the same language or will targeting the journal involve significant revision?
**Step 3: Revise the manuscript**
Read the journal author guidelines and scan recent papers to identify what needs to be edited, which might include
1. Voice: what disciplinary or professional voice does the journal publish in? Can you write in that voice? If not, consider drawing in a new co-author to support.
2. Structure: some journals want very short Introductions, others very long. Make the manuscript feel to the editor like ‘one of our papers’.
3. Fully follow the author guidelines. Delete any sections no longer needed. If there are new sections to complete (e.g., ‘What does this study contribute?’) then give them the same attention as the rest of the text received in its initial development.
4. Spelling: ensure to use British English (‘randomised’) or US English (‘randomized’) as per journal style, and consistently throughout. To supplement your careful proof-reading, set Language in Microsoft Word to ‘English (United Kingdom)’ or ‘English (United States)’ and check spelling.
5. Title: ensure your title looks like other recently-published titles from the target journal.
6. Abstract: this is central, and is the main component that many editors will read before deciding whether to reject or send for review. Ensure it reads like other abstracts of recent papers in the target journal.
7. Examples: ensure you know what country the journal is based in, which might be obvious from title (e.g., *Canadian Journal of Psychiatry*) or might not (e.g., *Epidemiology and Psychiatric Sciences* is Italian). Ideally include some examples relating to your topic from the journal’s country, or as a minimum if there are several examples ensure they do not all come from the same different country.
8. References: some journals have a maximum, which might involve significant re-shaping of the content. Ensure references are formatted per journal requirements, which is why you should be using bibliographic software. For the previous submission you may have included some tangentially-relevant references to papers in that journal—delete these. Ensure you have at least three references, including ideally the first citation, to papers published in the target journal in the last two years.
9. Document name: Ensure all traces of the previous journal submission are expunged—do not submit a file named ‘Study for BMJ.docx’ to the British Journal of Psychiatry! Ensure revised cover letter is specific to the new journal.
**Step 4: Re-submit to the new target journal**

## Data Availability

Not applicable.
